# Correction: Genetic polymorphisms of muscular fitness in young healthy men

**DOI:** 10.1371/journal.pone.0317856

**Published:** 2025-01-16

**Authors:** Tomas Venckunas, Hans Degens

The first author, Tomas Venckunas, is incorrectly noted as the corresponding author. The correct corresponding author is Hans Degens. Hans Degens’s email address is: h.degens@mmu.ac.uk.
